# The Genotypic Variability among Short-Season Soybean Cultivars for Nitrogen Fixation under Drought Stress

**DOI:** 10.3390/plants12051004

**Published:** 2023-02-22

**Authors:** Dilrukshi Kombala Liyanage, Davoud Torkamaneh, François Belzile, Parthiba Balasubramanian, Brett Hill, Malinda S. Thilakarathna

**Affiliations:** 1 Department of Agricultural, Food and Nutritional Science, University of Alberta, Edmonton, AB T6G 2P5, Canada; 2 Département de Phytologie, Université Laval, Québec City, QC G1V 0A6, Canada; 3 Institut de Biologie Intégrative et des Systèmes (IBIS), Université Laval, Québec City, QC G1V 0A6, Canada; 4 Agriculture and Agri-Food Canada, Lethbridge Research and Development Centre, Lethbridge, AB T1J 4B1, Canada

**Keywords:** soybean, drought, symbiotic nitrogen fixation, candidate genes, quantitative trait locus, genome wide association study

## Abstract

Soybean fixes atmospheric nitrogen through the symbiotic rhizobia bacteria that inhabit root nodules. Drought stress negatively affect symbiotic nitrogen fixation (SNF) in soybean. The main objective of this study was to identify allelic variations associated with SNF in short-season Canadian soybean varieties under drought stress. A diversity panel of 103 early-maturity Canadian soybean varieties was evaluated under greenhouse conditions to determine SNF-related traits under drought stress. Drought was imposed after three weeks of plant growth, where plants were maintained at 30% field capacity (FC) (drought) and 80% FC (well-watered) until seed maturity. Under drought stress, soybean plants had lower seed yield, yield components, seed nitrogen content, % nitrogen derived from the atmosphere (%Ndfa), and total seed nitrogen fixed compared to those under well-watered conditions. Significant genotypic variability among soybean varieties was found for yield, yield parameters, and nitrogen fixation traits. A genome-wide association study (GWAS) was conducted using 2.16 M single nucleotide single nucleotide polymorphisms (SNPs) for different yield and nitrogen fixation related parameters for 30% FC and their relative performance (30% FC/80% FC). In total, five quantitative trait locus (QTL) regions, including candidate genes, were detected as significantly associated with %Ndfa under drought stress and relative performance. These genes can potentially aid in future breeding efforts to develop drought-resistant soybean varieties.

## 1. Introduction

Soybean (*Glycine max* (L) Merr) is a main grain legume crop grown around the world, and is primarily used for oil, food, and feed production [1]. Soybean originated and was domesticated in China 6000–9000 years ago from *Glycine soja* (Sieb. & Zucc.) [2,3]. Soybean production has increased significantly over the last five decades as a result of the expansion of the growing areas [4]. In Canada, the highest amounts of soybean production were recorded in Ontario, followed by Quebec, Manitoba, the Maritimes, and Saskatchewan, and the total production in Canada accounts for 6.54 million metric tons (MMT) of soybean in 2022 [5].

It is predicted that drought stress will be one of the world’s costliest climatic problems in the near future [6]. The agricultural regions of the Canadian Prairies are particularly vulnerable to frequent drought conditions [7]. Drought stress limits soybean productivity and affects yield stability [8]. Soybean yield can decrease by more than half due to drought stress, resulting in significant losses for farmers [9]. Drought stress reduces grain legume productivity in all growth stages; however, drought stress during the reproductive and grain filling stages causes significant yield loss [10]. This yield loss is represented in different ways, such as a decrease in pod number, poorly developed pods, reduction in seed weight, reduction in seed number, and decline in seed quality [11]. The plant growth stage and the duration of drought stress are important in determining the level of impact in soybean [9,12]. Drought stress induced on early maturity soybean varieties at vegetative stages results in reduced plant height, decline in seed number at the early reproductive stages, and reduced seed weight at late reproductive stages [13]. The drought conditions between flowering and early seed filling stages can influence the vegetative growth of branches and lead to a decrease in seed yield in branches [14]. For example, research in China found that drought stress at the flowering and seed filling stages reduced yield by 73 and 82%, respectively [9]. Long-term drought stress during the reproductive stages reduces biomass allocation to reproductive organs and results in lower seed yield in soybean [15].

One of the most intriguing characteristics of soybean is its ability to form a symbiotic relationship with rhizobia bacteria in root nodules. These bacteria can convert atmospheric nitrogen into ammonia inside the root nodules and, in return, the host plant provides photosynthesis products for rhizobia metabolism [16]. Symbiotic nitrogen fixation (SNF) is sensitive to different biotic and abiotic factors [16,17] where the percentage of nitrogen derived from the atmosphere (%Ndfa) can vary from 0 to 95% in soybean due to these biotic and abiotic factors [1,16,18,19]. Among the different abiotic factors, drought stress is a major factor that limits SNF in soybean [16,20,21]. Drought stress affects different stages of legume-rhizobia symbiosis, such as root hair infection, nodule growth and development, and nodule function [22,23]. Furthermore, drought stress inhibits nitrogenase activity which is the key enzyme in catalyzing the reduction of dinitrogen (N_2_) to ammonia (NH_3_) [24]. Reduced SNF ultimately leads to reductions in grain yield, seed nitrogen, and grain protein production. Drought stress reduces SNF in nodules due to oxygen limitation in nodules, carbon scarcity, and nitrogen fixation feedback inhibition [25,26,27].

In the last decade, genome-wide association studies (GWAS) have become a feasible approach for discovering beneficial alleles from genetic diversity panels. GWAS have achieved extensive use in crops, despite being a relatively new tool in the fields of plant breeding and molecular biology. Different yield- and nitrogen fixation-related parameters have been considered in previous GWAS in soybean [28], including 100-seed weight [29,30,31,32,33], seed yield [29,30,31,32,34], number of pods [29,31], pod weight [33,34,35,36], yield stability [8], number of seeds per plant [31,37], number of seeds per pod [37], seed moisture content [33], and %Ndfa [38]. However, there is a research gap in identifying alleles involved in SNF in soybean under drought conditions.

The main objective of this study is to identify allelic variation associated with different yield parameters and SNF in soybean under drought stress and to identify the genomic regions controlling drought-tolerant SNF in short-season soybean varieties. A diversity panel of 103 Canadian short-season soybean cultivars was phenotyped for multiple yield- and nitrogen fixation-related traits, including the number of pods per plant, number of seeds per plant, seed yield, 100-seed weight, %Ndfa, seed nitrogen, and total seed nitrogen fixed under drought conditions to perform a GWAS analysis.

## 2. Materials and Methods

### 2.1. Germplasm, Plant Materials, and Growth Conditions

A diverse Canadian short-season soybean panel consisting of 103 soybean genotypes was used in this study (Table S1) [39]. First, seeds were surface sterilized using 70% ethanol for two minutes, and then washed five times with autoclaved double-distilled water [40]. The professional growing mix (Sun Gro Horticultural Canada Ltd., Seba Beach, AB, Canada) and sand (Target Products Ltd., Morinville, AB, Canada) were mixed in a 3:1 ratio in 6.52 L pots (H.J.S. Wholesale Ltd., Winnipeg, MB, Canada). Initially, three seeds were planted in a pot. Plants were maintained in a greenhouse with supplemental lighting (range: 500–600 mol m^−2^ s^−1^ at the top of the canopy, Fortimo LED Line, High Flux VO) at 26 ± 2 °C during the day and 20 ± 2 °C at night. The photoperiod was kept at 16/8 h light/dark cycles. Extra plants were removed, leaving one plant per pot after one week of plant growth. Seedlings were inoculated with 2 mL of *Bradyrhizobium japonicum* USDA 110 inoculum (rhizobial density OD_600_ = 0.1) [40]. The same process was repeated one week after the first inoculation to ensure successful nodulation. Each week, plants received 100 mL of quarter-strength N-free Hoagland’s nutrient solution (HOP03-50LT, Caisson Labs, Smithfield, UT, USA). Plants were labeled with 25 mL of 0.5 mM K^15^NO_3_ solution (10 atom% ^15^N; 348481-25G; Sigma Aldrich, Oakville, ON, Canada) two and three weeks after planting to measure SNF.

### 2.2. Field Capacity of Growth Media

The determination of field capacity of growth media was completed in a separate study. First, the bottom of the 6.52 L pots was covered with a coffee filter (12” Mother Parkers Coffee Filters) to avoid any leakage of potting mixture. Then, pots were filled with a mixture of sand (Target Products Ltd., Morinville, AB, Canada) and growing mix (Sun Gro Horticultural Canada Ltd., Seba Beach, AB, Canada) on a 1:3 volume basis till a constant final weight was obtained (e.g., 4500 g). The initial dry weight of the soil (D_w_) was measured after drying in an oven at 80 °C until a constant weight was obtained [41]. The pots were watered until the growth media was saturated and water drained out from the bottom. The top of the pots was covered using aluminum foil to avoid evaporation. The pots were undisturbed for 24 h until no further water drainage was observed, and then the final saturated weight was recorded (Sw). The field capacity (FC) was calculated as FC = S_W_ − D_W_. Accordingly, final weights for the well-watered treatment (80% FC) and drought treatment (30% FC) were calculated [42].

### 2.3. Drought Treatment and Yield Data Collection

After three weeks of germination, the soil moisture content of the pots was maintained at 80% FC (well-watered) and 30% FC (drought). The 30% FC was reached by withholding water until pots reached 30% FC. This moisture adjustment in all the pots was carried out using an Arduino-based, semi-automated irrigation system throughout the greenhouse experiment [42]. The main treatments were genotypes and moisture levels, which were allocated according to a randomized complete block design with four replicates per treatment (n = 4). Different yield parameters such as number of pods per plant, number of seeds per plant, 100-seed weight, and seed weight per plant were collected at seed maturity.

### 2.4. Determination of Nitrogen Fixation-Related Parameters

The %Ndfa was measured using the isotope dilution method [43]. Seeds were oven-dried at 60°C for three days and ground to a coarse powder by using a coffee grinder. A subsample from each sample was further ground in a small Eppendorf tube along with a steel bead in a bead beater homogenizer (OMNI International, Kennesaw, GA, USA). Then, a 5 mg of soybean powder sample was measured into a small tin capsule (8 mm × 5 mm, D1008, Isomass Scientific Inc., Calgary, AB, Canada) using a microbalance. Samples were enveloped and compressed into a tiny pellet to make sure no air remained. The tin capsules were arranged in a 96-well plate and sent to the Stable Isotope Facility, Agriculture and Agri-Food Canada, Lethbridge Research and Development Centre to analyze ^15^N and total N% [44]. The encapsulated seed samples were analyzed with a Finnigan Delta V Plus (Thermo Electron, Bremen, Germany) Isotope Ratio Mass Spectrometer (IRMS) fitted with a Flash 2000 Elemental Analyzer (Thermo Fisher Scientific, Voltaweg, The Netherlands) and a Conflo IV interface (Thermo Fisher Scientific, Bremen, Germany) between the IRMS and the analyzer. The isotope standards were L-glutamic acid (USGA40) and L-glutamic acid enriched in ^15^N (USGA41A) (United States Geological Survey). The %Ndfa of the soybean was calculated using the following formula according to the isotope dilution technique:

$$\%Ndfa=\left(1-\frac{{\mathrm{atom}\%\;}^{15}N\;excess_{\left(fixing\;plant\right)}}{{\mathrm{atom}\%\;}^{15}N\;excess_{\left(non-fixing\;plant\right)}}\right)\times 100$$

where atom % ^15^N excess = atom % ^15^N soybean—0.3663. The amount of seed nitrogen derived from nitrogen fixation was calculated based on the total seed nitrogen content and %Ndfa (seed N content × %Ndfa/100).

### 2.5. Statistical Analysis

Analysis of variance (ANOVA) set at α < 0.05 was used to examine the effects of moisture treatments and soybean genotypes. A two-factor factorial design was used to analyze the data. The main factor was the soil moisture level with two levels of soil moisture: 80% FC (well-watered) and 30% FC (drought), and the subfactor was soybean genotypes. R 3.5 was used to perform the ANOVA, frequency distributions, and Pearson correlations among different response variables [45].

### 2.6. Genotyping Data

The whole population was genotyped previously by using genotyping-by-sequencing (GBS) and whole-genome-sequencing (WGS) approaches [39,46,47] with 56 samples in common. Briefly, the SNPs were called using the Fast-GBS and Fast-WGS pipelines for GBS and WGS data, respectively [47,48], using soybean Williams 82 reference genome (Gmax_275_Wm82.a2.v1) [49]. These analyses resulted in two datasets of 56 K (GBS) and 4.3 M (WGS) SNPs. A missing genotype imputation using WGS dataset as a reference panel was previously performed using BEAGLE v4.1 [50] as described by Torkamaneh and Belzile 2021 [39,51]. The genotyping quality control and filtering measures were extensively described by Malle et al., 2020 [39]. Here, we extracted genotypic data for 103 samples from this larger collection (137 samples) and used VCFtools [52] to retain SNPs with minor allele frequency (MAF) ≥0.05 and heterozygosity ≤0.1 that resulted in a panel of 2.16 M SNPs.

### 2.7. Population Structure

In the panel of 2.18 M SNPs, LD-based pruning (r^2^ > 0.5) was done with PLINK [53] to get a reduced and uniformly distributed set of 14 K markers. The fastSTRUCTURE algorithm [54] was used to characterize the population structure using tested subpopulations (K) from 1 to 13 with three independent runs of each. The python ‘Choseek.py’ script was used to find the most suitable K value based on the rate of change in LnP between the successive K values. In addition, a phylogenetic tree was constructed in TASSEL 5.0 using the Neighbor-Joining method (Supplementary Figure S1), and a scree plot (Supplementary Figure S2) was used to evaluate the most informative Principal Components (PCs) (Supplementary Figures S3 and S4). Furthermore, a Kinship matrix was calculated using the efficient mixed-model association (EMMA) method.

### 2.8. Genome-Wide Association Study

A GWAS was carried out with 2.16 M SNPs utilizing the fixed and random model circulation probability unification (FarmCPU) model [55] implemented in rMVP package on Microsoft R Open [56]. To reduce false positives, the population matrix (Q) and Kinship Matrix (K) were measured and used as covariates. A genome-wide significance threshold level that is less than 0.05 was used to find significant associations using the false discovery rate (FDR) test [57].

### 2.9. Candidate Gene Identification

The soybean public database SoyBase 2020 [58] and soybean reference genome annotation were used to identify candidate genes for the yield- and nitrogen-fixation-related parameters. The quantitative trait loci (QTL) flanking areas were set to 100 kb on either side of the QTL peak to seek potential genes involved in yield parameters. The tool ePlant2 was used to get more information about the gene expression in different tissues [59].

## 3. Results

### 3.1. Phenotypic Variation of Yield- and Nitrogen Fixation-Related Traits in Soybean

**Figure 1:**
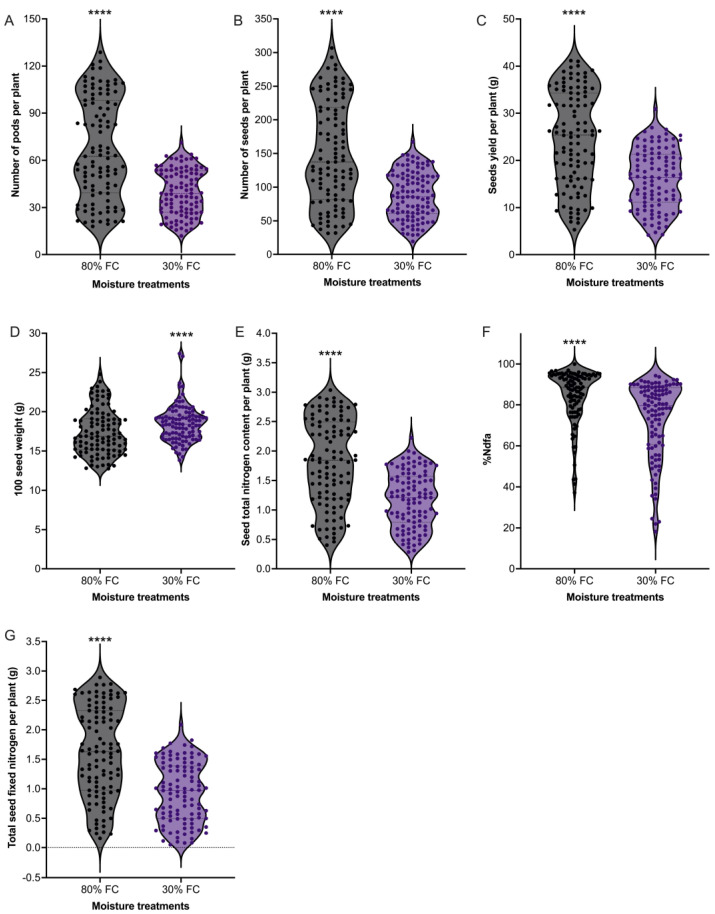
Distribution of yield- and SNF-related traits under well-watered and drought conditions in different soybean cultivars. (**A**) Number of pods per plant. (**B**) Number of seeds per plant. (**C**) Seed yield per plant. (**D**) 100-seed weight. (**E**) Seed total nitrogen content per plant. (**F**) Percentage nitrogen derived from the atmosphere (%Ndfa). (**G**) Total seed nitrogen fixed per plant. Each data point represents the mean value of a single cultivar. The asterisks (****) indicate significant differences between the 80% field capacity (FC) and 30% FC at *p* < 0.0001.

A significant phenotypic variation among 103 soybean genotypes was found for the number of pods per plant, the number of seeds per plant, seed yield, 100-seed weight, %Ndfa, seed total nitrogen content, and total seed nitrogen fixed (*p* < 0.0001) (Figure 1). The soil moisture content also had a significant effect on the yield parameters, grain yield, and SNF-related traits (Figure 1). In comparison to the well-watered treatment, drought stress significantly reduced the number of pods per plant (67.2 vs. 39.6) (Figure 1A), the number of seeds per plant (150.0 vs. 87.9) (Figure 1B), and grain yield (24.7 vs. 16.1 g) (Figure 1C). However, 100-seed weight was higher under 30% FC (18.5 g) compared to the well-watered 80% FC treatment (17.5 g) (Figure 1D). Regarding the 80% FC well-watered treatment, drought treatment reduced the seed total nitrogen content (1.8 vs. 1.2 g N plant^−1^) (Figure 1E), %Ndfa (84.4 vs. 73.1%) (Figure 1F), and total seed nitrogen fixed (1.6 vs. 0.9 g N plant^−1^) (Figure 1G).

### 3.2. Correlations among Yield- and Nitrogen Fixation-Related Traits in Soybean

**Figure 2:**
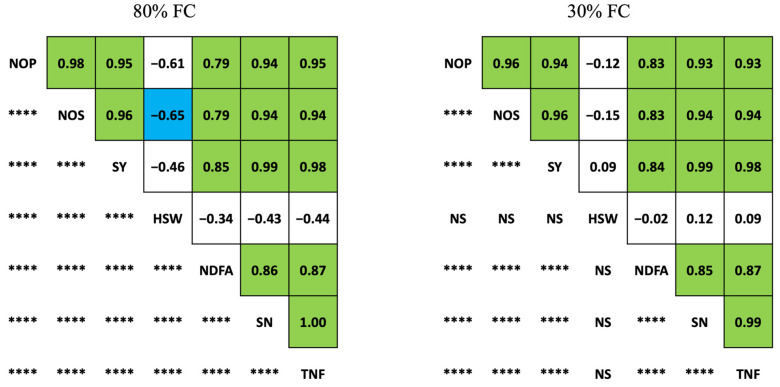
Correlations among yield- and nitrogen fixation-related traits in soybean under drought and well-watered conditions. Numbers above the diagonal correspond to Pearson’s correlation coefficients (r). Green boxes highlight the positive values exceeding 0.65 and blue boxes highlight the negative values exceeding or equal to 0.65 [60]. Below the diagonal show the degree of significance of the corresponding correlations between traits (**** *p* < 0.0001, and NS: not significant). FC, field capacity; NOP, number of pods; NOS, number of seeds; SY, seed yield; HSW, 100-seed weight; %Ndfa, percentage of nitrogen derived from the atmosphere; SN, seed nitrogen; TNF, total nitrogen fixed.

Significant correlations among different plant traits were found under the 80% FC and 30% FC treatments (Figure 2). The %Ndfa and total nitrogen fixation (g N plant^−1^) were positively correlated with the number of pods (r = 0.79, 0.83), number of seeds (r = 0.79, 0.83), seed yield (r = 0.85, 0.84), and seed nitrogen content (r = 0.86, 0.85) under both 80% FC and 30% FC treatments. Seed yield was positively correlated with the number of pods (r = 0.95, 0.94), number of seeds (r = 0.96, 0.96), %Ndfa (r = 0.85, 0.84), seed nitrogen content (r = 0.99, 0.99), and total nitrogen fixed (r = 0.98, 0.98) under 80% FC and 30% FC treatments. Interestingly, the number of pods, number of seeds, seed yield, %Ndfa, seed nitrogen, and total seed nitrogen fixed were not correlated with 100-seed weight under the 30% FC treatment. The number of seeds was negatively correlated with 100-seed weight (r = −0.65) under the 80% FC treatment.

### 3.3. Genome-Wide Association of Yield- and Nitrogen Fixation-Related Traits

**Figure 3:**
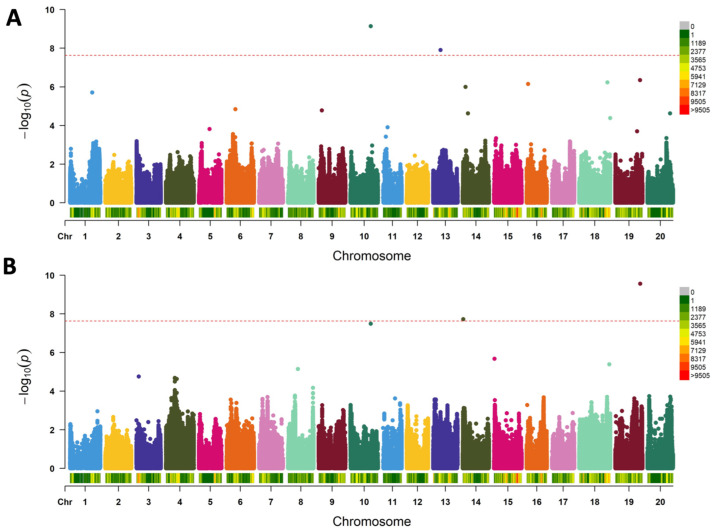
Manhattan plots showing significantly associated SNPs detected in genome-wide association results for percentage nitrogen derived from the atmosphere (%Ndfa) under (**A**) %Ndfa—30% field capacity (FC); (**B**) %Ndfa—relative performance. Relative performance was calculated as the ratio of %Ndfa under 30% FC vs. 80% FC. Negative log_10_ (*p*-values, *y*-axis) describing the strength of the association between each marker and trait are plotted against the physical position of each marker (*x*-axis). The pink dashed line indicates the significance threshold (FDR = 5%) and beyond that are considered as significant associations. Each colored dot represents a SNP.

As reported in Seck et al., 2020, both GBS and WGS genotyping techniques were utilized in this study to cover the entire soybean genome. A subset of 14 K trimmed SNPs was used to characterize population structure. The optimal number of subpopulations (K) was six to nine, and confirmed with both principal component analysis (PCA) and phylogeny analysis. An extensive genomic SNP coverage for the panel of 103 soybean genotypes was obtained with almost 1 SNP every 455 bp (Supplementary Figure S5), perfectly suitable for GWAS analysis. GWAS analyses were performed for yield- and nitrogen fixation-related traits, and yield parameters under 30% FC and their relative performance (30% FC/80% FC) using 2.16 M SNPs and the FarmCPU statistical model. We found two SNPs were associated with %Ndfa-qNDFA-30-1, qNDFA-30-2 in 30% FC (Figure 3A), and another three SNPs were associated with %Ndfa—qNDFA-RP-1, qNDFA-RP-2, qNDFA-RP-3 in relative performance (Figure 3B) (Table 1).

### 3.4. Yield- and Nitrogen Fixation-Trait-Related Candidate Genes

All the genes that are residing in whole or in part within the five QTLs of interest were extracted from SoyBase. Table 2 provides the complete information of these genes, including their annotations. Based on their annotation, we identified some strong candidate genes separately for 30% FC and their relative performance as mentioned below. We found four strong candidate genes for qNDFA-30-1, three for qNDFA-RP-1, and two for qNDFA-RP-3 based on the literature review and previous studies.

Candidate genes under drought stress:

For the trait %Ndfa, we found the gene *Glyma.10g144600* which is annotated for Glycogen synthase kinase-3 and Glycogen synthase kinase (qNDFA-30-1) (Table 2). The gene *Glyma.10g145300* that encodes for galactinol synthase was also found as a candidate gene for qNDFA-30-1. The gene *Glyma.10g144300* annotated for S-adenosylmethionine synthetase 2 was also found in this region (qNDFA-30-1).

Candidate genes under relative performance:

For the relative performance of %Ndfa, we found the gene *Glyma.06g197700* which is annotated for putative endonuclease or glycosyl hydrolase with C2H2-type zinc finger domain (qNDFA-RP-1) (Table 2). The gene *Glyma.06g198600* encodes for Ankyrin repeat family protein identified in this region. Moreover, the *Glyma.06g199700* gene encoding for Remorin family protein was recognized as an important candidate gene (qNDFA-RP-1). The gene *Glyma.19g212800* annotated as sucrose synthase 3 is another strong candidate gene for qNDFA-RP-3 (Table 2). The gene *Glyma.19g213900* encodes for drought-responsive family protein was also identified for this trait.

## 4. Discussion

### 4.1. Significant Phenotypic Variation of Yield- and Nitrogen Fixation-Related Traits in Soybean

As previously stated, we discovered significant phenotypic variability for several yield- and nitrogen fixation-related parameters at the maturity stage. Our findings corroborate with previous findings, wherein phenotypic variability for different yield- and nitrogen fixation-related traits were found for number of pods [29], number of seeds [37], seed weight [34,61], 100-seed weight [29,30,62,63,64], and %Ndfa [38]. The presence of phenotypic variation within a germplasm pool for various yield- and nitrogen fixation-related traits is critical for plant breeders to make breeding selections.

Under drought stress, varieties such as DH 748, OAC Lakeview, Mario, OAC Madoc, and OAC Champion produced the most pods per plant, whereas Albions, 9004, Auriga, OAC-07-04C, and AC 2001 produced the least. In terms of the number of seeds per plant, DH 748, OAC Avatar, OAC Ginty, OAC Madoc, and OAC Wallace had the most, while Albinos, Auriga, 9004, Maple Donovan, and AC 2001 had the fewest under drought stress. Seed yield was highest in DH 748, OAC Avatar, OAC Ginty, OAC-09-35C, and OAC Lauralain, and lowest in Albions, Maple Donovan, 9004, Auriga, and Naya under drought. The 100-seed weight was greater in DH 618, Alta, Amasa, DH 420, and Ohgata under drought stress, and the lower in Maple presto, 90B11, Maple Donovan, 90A01, and OAC Madoc.

All the soybean cultivars were ranked based on their %Ndfa and grain yield under 30% FC (Table S2). In terms of nitrogen fixation-related traits, OAC Champion, DH 748, OAC Oxford, Toki, and OAC Wallace had the highest %Ndfa, while Maple Donovan, Costaud, Albinos, Gaillard, and Naya had the lowest under drought stress (Table S2). The soybean cultivars, which ranked higher for %Ndfa, also ranked for higher grain yield and vice versa under drought conditions (Table S2). The nitrogen content of seed was highest in DH 748, OAC Ginty, OAC Avatar, OAC Oxford, and 91M10 under drought stress, whereas Maple Donovan, 9004, Albinos, Auriga, and AC 2001 had the lowest. Total seed nitrogen fixation was highest in DH 748, OAC Ginty, OAC Avatar, OAC Oxford, and OAC Stratford, while Maple Donovan, Costaud, Albinos, 9004, and Gaillard were lowest under drought stress.

### 4.2. Drought Stress on Symbiotic Nitrogen Fixation

In this study, drought stress had significant negative impact on symbiotic nitrogen fixation in soybean. Drought stress reduced %Ndfa by 13.4%, total seed nitrogen by 34.9%, and the amount of seed nitrogen fixed by 42.1% compared to the well-watered plants. The reduction in nitrogen fixation under drought stress may be due to multiple plant responses. Sucrose is the primary carbon source supplied from shoots to bacteroids to fuel the symbiotic nitrogen fixation process. Sucrose synthase hydrolyzes sucrose into hexose and then it catabolizes into phosphoenolpyruvate (PEP) through the glycolytic pathway, which is further converted to oxaloacetate by PEP carboxylase (PEPC) [22]. Oxaloacetate is reduced to malate by malate dehydrogenase (MDH) regenerating NAD+ [65]. During drought stress, nodule sucrose synthase activity sharply declines [66], hence limiting the carbon flux required for bacteroid respiration. Drought stress directly affects nodule activity due to increased oxygen diffusion resistance and decreased nitrogenase enzyme activities, thereby affecting the metabolic activities of nitrogen-fixing rhizobia [21]. Furthermore, feedback inhibition of nitrogen fixation can also take place due to increased ureides and free amino acids in soybean plant tissues [22,67].

### 4.3. Correlations among Yield- and Nitrogen Fixation-Related Traits

Many yield- and nitrogen fixation-related traits were found to be highly and significantly correlated. These findings are also consistent with previous literature. For instance, it has been observed that the number of pods per plant has a significant correlation between the number of seeds per pod and seed yield [29,68,69,70]. We found a negative correlation between the number of seeds and 100-seed weight. The negative relationship could be a result of the seed size decreasing when the number of seeds per plant increases [69,70]. Soybean seeds contain a significant amount of plant nitrogen, (~71%) [71] and it mainly derives from SNF. The strong positive correlation between the %Ndfa and seed nitrogen content under drought conditions indicates that symbiotic nitrogen fixation is crucial for seed nitrogen accumulation and protein production in drought-stressed soybean.

### 4.4. Genome-Wide Association Using Whole-Genome Data Revealed Five QTLs Controlling %Ndfa

In this study, we discovered five genomic regions, or QTLs, that contribute to %Ndfa under drought stress and relative performance. In the same manner, some previous GWAS in soybean have also revealed loci for %Ndfa [38]. We discovered that the majority of the QTL regions identified in our study are novel. Furthermore, the majority of GWAS in soybean were based on seed yield because this is a key trait for crop improvement. A limited number of GWAS have been conducted for the %Ndfa in soybean, specifically under drought conditions.

### 4.5. Candidate Genes for %Ndfa-Associated QTLs

Candidate genes under drought stress:

The gene *Glyma.10g144600* associated with %Ndfa (qNDFA-30-1) is a strong candidate gene annotated for glycogen synthase kinase-3 and was highly expressed in roots (Table 2). Under salt stress, the glycogen synthase kinase 3 (GSK3)-like kinase plays an important role in inhibiting symbiotic signaling and nodule formation in soybean [72,73,74]. The GSK3-like kinases directly regulate the activities of *G. max* Nodulation Signaling Pathway 1 (*GmNSP1*) proteins, facilitating legume-rhizobium symbiosis under salt stress [72]. Galactinol and raffinose act as osmoprotectants for drought-stress tolerance in plants [75] and the gene *Glyma.10g145300,* annotated for galactinol synthase, was highly expressed in roots and identified for qNDFA-30-1 (Table 2). Overexpression of galactinol synthase—which catalyzes the first step in the biosynthesis of raffinose family oligosaccharides—results in increased galactinol and raffinose accumulation and improved drought tolerance in soybean [75,76]. It is found that the gene *Glyma.10g144300*-S-adenosylmethionine synthetase 2 is down regulated in soybean root tips and roots under drought conditions, and it is highly expressed in soybean nodules (qNDFA-30-1) [77] (Table 2).

Candidate genes under relative performance:

The gene *Glyma.06g197700,* annotated for putative endonuclease or glycosyl hydrolase with C2H2-type zinc finger domain, was found for the relative performance of qNDFA-RP-1 (Table 2). It is found that in soybean, C2H2 zinc finger proteins are involved in nodule development, nodule function, and nodule signal transduction [78]. The gene *Glyma.06g198600* encodes for Ankyrin repeat family protein (ANK) and was found in the same region (qNDFA-RP-1) (Table 2). It has been found that the overexpression of *GmANK114* improved the survival rate of transgenic soybean hairy roots under drought and salt stresses [79]. *GmANK114* overexpression in soybean hairy root showed higher proline, lower malondialdehyde contents, and lower H_2_O_2_ and O^2−^ contents in response to drought or salt stress [79]. The gene *Glyma.06g199700* encodes for Remorin family protein and is highly expressed in soybean roots (qNDFA-RP-1) (Table 2). Remorin participates in a wide range of biotic and abiotic stresses, and root nodule development [80]. *GmREM1.1*, for example, was found to be highly expressed in the nodule primordia and the inner cortex region of root nodules. Furthermore, *GmREM2.1* transcription was mostly found in rhizobia-infected cells [80]. The gene *Glyma.19g212800,* annotated as sucrose synthase 3, was found in qNDFA-RP-3 (Table 2). Sucrose synthase plays a key role in the regulation of nodule carbon metabolism [23,66,81]. Therefore, this gene will directly affect SNF in soybean, as photosynthesis and carbon supply are hampered under the drought stress. The gene *Glyma.19g213900*, which encodes for drought-responsive family protein, was found in the same QTL region (Table 2). These drought-responsive family genes are differentially expressed upon exposure to drought stress [82,83]. Importantly, drought-responsive candidate genes contribute to the development of drought-tolerant soybean cultivars [83].

### 4.6. Limitations

There are some limitations associated with the current study. This study was conducted under controlled environmental conditions, where plants were grown in pots using peat-sand-based media with two moisture levels (30% FC and 80% FC). These conditions may not precisely mimic the drought conditions that occur under field conditions.

## 5. Conclusions

This study found significant genotypic variability among soybean genotypes in terms of the number of pods per plant, the number of seeds per plant, seed weight, 100-seed weight, %Ndfa, seed nitrogen, and total nitrogen fixed. The GWAS conducted for this research revealed five QTLs for %Ndfa under drought conditions and relative performance. Furthermore, strong candidate genes were discovered to support the findings. The current study will contribute toward understanding the genetics underlying different yield- and nitrogen fixation-related traits and drought tolerance in soybean.

## Figures and Tables

**Table 1:** List of quantitative trait loci (QTL) associated with percent nitrogen derived from the atmosphere (%Ndfa) under 30% field capacity (FC) and relative performance.

Moisture Effect	Chr Number	MSS Position	QTL ID	Minor Allele Frequency	*p* Value	Effect
30% FC	10	37,995,110	qNDFA-30-1	0.19	<0.0001	−6.87
	13	13,866,995	qNDFA-30-2	0.29	<0.0001	−4.79
Relative	6	18,244,365	qNDFA-RP-1	0.06	<0.0001	−0.12
Performance	14	994,141	qNDFA-RP-2	0.12	<0.0001	−0.05
	19	46,575,733	qNDFA-RP-3	0.11	<0.0001	−0.09

FC, field capacity; Chr, Chromosome; MSS, Most Significant SNP. Relative performance was calculated as the ratio of %Ndfa under 30% FC vs. 80% FC.

**Table 2:** Candidate genes associated with drought stress (30% field capacity) and relative performance.

Treatment	Chr Number	MSS Position	REF/ALT	QTL ID	Candidate Genes	Orthologous Genes in *Arabidopsis*	Annotations
30% FC	10	37,995,110	G/A	qNDFA-30-1	Glyma.10g144600	Gene Model: AT5G26751.1	Glycogen synthase kinase—FJ460228
					Glyma.10g144600	Gene Model: AT5G26751.1	Glycogen synthase kinase-3—BT093874
					Glyma.10g145300	Gene Model: AT2G47180.1	Galactinol synthase 1- AK245720, AY126715
					Glyma.10g144300	Gene Model: AT4G01850.1	S-adenosylmethionine synthetase 2
Relative Performance	6	18,244,365	G/A	qNDFA-RP-1	Glyma.06g197700	Gene Model: AT5G61190.1	Putative endonuclease or glycosyl hydrolase with C2H2-type zinc finger domain
					Glyma.06g198600	Gene Model: AT2G03430.1	Ankyrin repeat family protein
					Glyma.06g199700	Gene Model: AT5G61280.1	Remorin family protein
	19	46,575,733	C/T	qNDFA-RP-3	Glyma.19g212800	Gene Model: AT4G02280.1	Sucrose synthase 3
					Glyma.19g213900	Gene Model: AT4G02200.1	Drought-responsive family protein

FC, field capacity; Chr number, Chromosome number; MSS, Most significant SNP. Relative performance was calculated as the ratio of %Ndfa under 30% FC vs. 80% FC.

## Data Availability

The data presented in this study (mean values) are available in Supplementary File S1.

## Supplementary Materials

The following supporting information can be downloaded at: https://www.mdpi.com/article/10.3390/plants12051004/s1, Figure S1: Phylogenetic tree constructed using a core set of 103 Canadian short-season soybean lines in TASSEL 5.0; Figure S2: Scree plot created for genotypic data using 10 principal components. The *y*-axis is the eigenvalue, and the *x*-axis is the ten principal components; Figure S3: Principal Component Analysis (PCA) scatter plot for the genotypic data using the principal component 1 (PC1) and principal component 2 (PC2). The *y*-axis is the PC1 and the *x*-axis is the PC2; Figure S4: Principal Component Analysis (PCA) scatter plot for the genotypic data using the principal component 1 (PC1) and principal component 3 (PC2). The *y*-axis is the PC1 and the *x*-axis is the PC3; Figure S5: Distribution of SNP markers across the soybean genome. Chromosomes appear horizontally with the density of SNPs depicted in the scale shown to the right; Table S1: The list of 103 short season soybean genotypes used in the study; Table S2: Ranking of soybean cultivars based on the percentage nitrogen derived from the atmosphere (%Ndfa) and grain yield under 30% field capacity.
