# Supplementary material for: The Genotypic Variability among Short-Season Soybean Cultivars for Nitrogen Fixation under Drought Stress
Source: Plants (Basel). 2023 Feb 22;12(5):1004. doi: 10.3390/plants12051004 (PMC10005650; doi:10.3390/plants12051004)
Supplement: Supplementary file 1 [file plants-12-01004-s001.zip › Supplementary Table S1.pdf]

Supplementary Table S1. The list of 103 short season soybean genotypes used in the study

9004	Casino	Lotus	OAC 09-01C	OAC Wallace
9063	Colby	Madison	OAC 09-17C	OAC Walton
90A01	Costaud	Mandarin	OAC 09-22C	OAC Woodstock
90A07	Dares	Maple Amber	OAC 09-35C	Ohgata
90B11	Delta	Maple Arrow	OAC Avatar	OT09-03
91M10	DH420	Maple Belle	OAC Ayton	OT11-01
AC 2001	DH530	Maple Donovan	OAC Bayfield	Phoenix
AC Brant	DH618	Maple Glen	OAC Carman	Roland
AC Bravor	DH748	Maple Isle	OAC Champion	S03-W4
AC Glengarry	Dundas	Maple Presto	OAC Clinton	Toki
AC Harmony	Evans	Maple Ridge	OAC Drayton	Venus
AC Hercule	Flambeau	Mario	OAC Ginty	Victoria
AC Orford	Gaillard	McCall	OAC Gretna	OAC Elora
AC Proteina	Gentleman	Misty	OAC Kent	OAC Perth
AC Proteus	Heather	Naya	OAC Lakeview	OAC Stratford
Albinos	Jari	OAC 01-26	OAC Lauralain	PS 36
Alta	Jutra	OAC 07-04C	OAC Madoc	QS5030.46Bp
Altesse	Kamichis	OAC 07-06C	OAC Morris	SECAN 07-27C
Altona	Katrina	OAC 07-26C	OAC Oxford	
Amasa	KG 41	OAC 08-11C	OAC Petrel	
Auriga	Korada	OAC 08-21C	OAC Prudence	
Bloomfield	Krios	OAC 08-22C	OAC Purdy	
