# Supplementary material for: The Genotypic Variability among Short-Season Soybean Cultivars for Nitrogen Fixation under Drought Stress
Source: Plants (Basel). 2023 Feb 22;12(5):1004. doi: 10.3390/plants12051004 (PMC10005650; doi:10.3390/plants12051004)
Supplement: Supplementary file 1 [file plants-12-01004-s001.zip › Supplementary Table S2.pdf]

Supplementary Table S2

Ranking of soybean cultivars based on the percentage nitrogen derived from the atmosphere (%Ndfa) and grain yield under 30% field capacity.

Soybean Cultivars	%Ndfa	Rank	Grain yield (g plant^{-1})	Rank
OAC Champion	94.33	1	23.06	16
DH748	93.76	2	30.87	1
OAC Oxford	92.39	3	25.30	6
Toki	92.32	4	20.02	34
OAC Wallace	91.93	5	24.95	7
Victoria	91.51	6	24.31	12
OAC Ginty	91.33	7	26.56	3
OAC Stratford	91.22	8	24.28	13
S03-W4	90.92	9	20.42	31
OAC Kent	90.84	10	21.51	25
Roland	90.50	11	20.64	28
OAC Gretna	90.30	12	22.46	21
SECAN 07-27C	90.25	13	20.59	30
OAC Bayfield	90.15	14	22.96	17
OAC Lakeview	90.11	15	24.03	15
OAC Avatar	90.10	16	26.94	2
OAC Clinton	90.00	17	18.88	39
OAC 08-22C	89.85	18	19.83	35
OAC Woodstock	89.55	19	24.07	14
OAC Purdy	89.09	20	20.63	29
Jutra	88.95	21	24.95	8
OAC Madoc	88.87	22	22.10	22
OAC Perth	88.83	23	24.66	9
PS 36	88.81	24	22.95	18
Venus	88.76	25	22.67	20
OAC Drayton	88.49	26	22.73	19
QS5030.46Bp	87.53	27	20.34	32
OAC Ayton	87.21	28	21.51	24
OAC Morris	87.09	29	24.34	11
90B11	87.03	30	11.13	78
Krios	86.90	31	19.38	37
OAC 01-26	86.85	32	16.38	52
DH530	86.31	33	21.64	23
Phoenix	86.29	34	19.40	36
OAC Petrel	85.65	35	17.60	45
OT11-01	85.52	36	17.15	46
91M10	85.16	37	24.59	10
OAC 09-35C	85.08	38	26.10	4
AC Bravor	84.76	39	20.10	33
AC Proteus	84.75	40	8.45	91
OAC Lauralain	84.13	41	25.63	5

OAC Walton	83.65	42	21.32	26
Mario	83.19	43	21.00	27
Kamichis	82.75	44	16.94	48
OAC 08-21C	82.18	45	18.10	42
Katrina	81.29	46	16.25	53
Maple Arrow	80.60	47	13.68	63
OAC Carman	80.53	48	15.63	57
Evans	80.34	49	18.38	40
Maple Amber	80.33	50	8.65	89
Amasa	79.62	51	17.93	43
Dares	79.59	52	17.05	47
Madison	78.40	53	16.80	49
Delta	78.39	54	17.75	44
OAC Prudence	78.29	55	15.72	56
OAC Elora	78.14	56	14.17	60
Misty	78.00	57	18.88	38
Maple Presto	77.43	58	7.51	94
OAC 07-26C	76.97	59	15.23	58
Colby	76.60	60	16.58	50
Jari	75.68	61	14.73	59
Casino	75.23	62	13.77	62
AC Hercule	74.91	63	11.76	74
Flambeau	74.86	64	8.73	88
AC Proteina	73.23	65	14.10	61
Lotus	72.86	66	11.78	73
Dundas	72.83	67	16.23	54
OAC 08-11C	71.57	68	18.28	41
Ohgata	70.52	69	16.49	51
AC Brant	69.51	70	13.40	65
DH420	68.01	71	11.53	77
KG 41	66.50	72	12.10	71
Maple Glen	65.50	73	12.90	68
OT09-03	64.95	74	12.60	70
OAC 09-22C	64.61	75	16.20	55
AC Glengarry	64.58	76	13.48	64
OAC 09-17C	61.66	77	12.68	69
DH618	60.66	78	11.58	76
90A01	60.26	79	9.36	84
OAC 07-06C	59.95	80	12.93	67
Mandarin	58.94	81	11.63	75
Altona	58.73	82	11.83	72
Heather	55.56	83	8.03	92
Altesse	55.43	84	12.93	66
OAC 07-04C	54.39	85	6.73	96
Maple Isle	53.38	86	9.41	83
90A07	53.10	87	8.95	87
AC Orford	51.67	88	10.33	81
Gentleman	50.18	89	9.13	86

9063	49.99	90	9.57	82
Maple Ridge	48.97	91	9.25	85
Bloomfield	48.01	92	7.73	93
Auriga	45.96	93	5.69	100
AC 2001	43.38	94	6.23	98
McCall	41.74	95	10.57	79
Alta	39.33	96	10.39	80
Maple Belle	36.35	97	8.55	90
9004	35.73	98	4.75	101
Naya	34.22	99	5.95	99
Gaillard	24.50	100	7.45	95
Albinos	23.04	101	4.18	103
Costaud	22.00	102	6.45	97
Maple Donovan	18.23	103	4.28	102
