# Supplementary figures and images for: The Genotypic Variability among Short-Season Soybean Cultivars for Nitrogen Fixation under Drought Stress
Source: Plants (Basel). 2023 Feb 22;12(5):1004. doi: 10.3390/plants12051004 (PMC10005650; doi:10.3390/plants12051004)

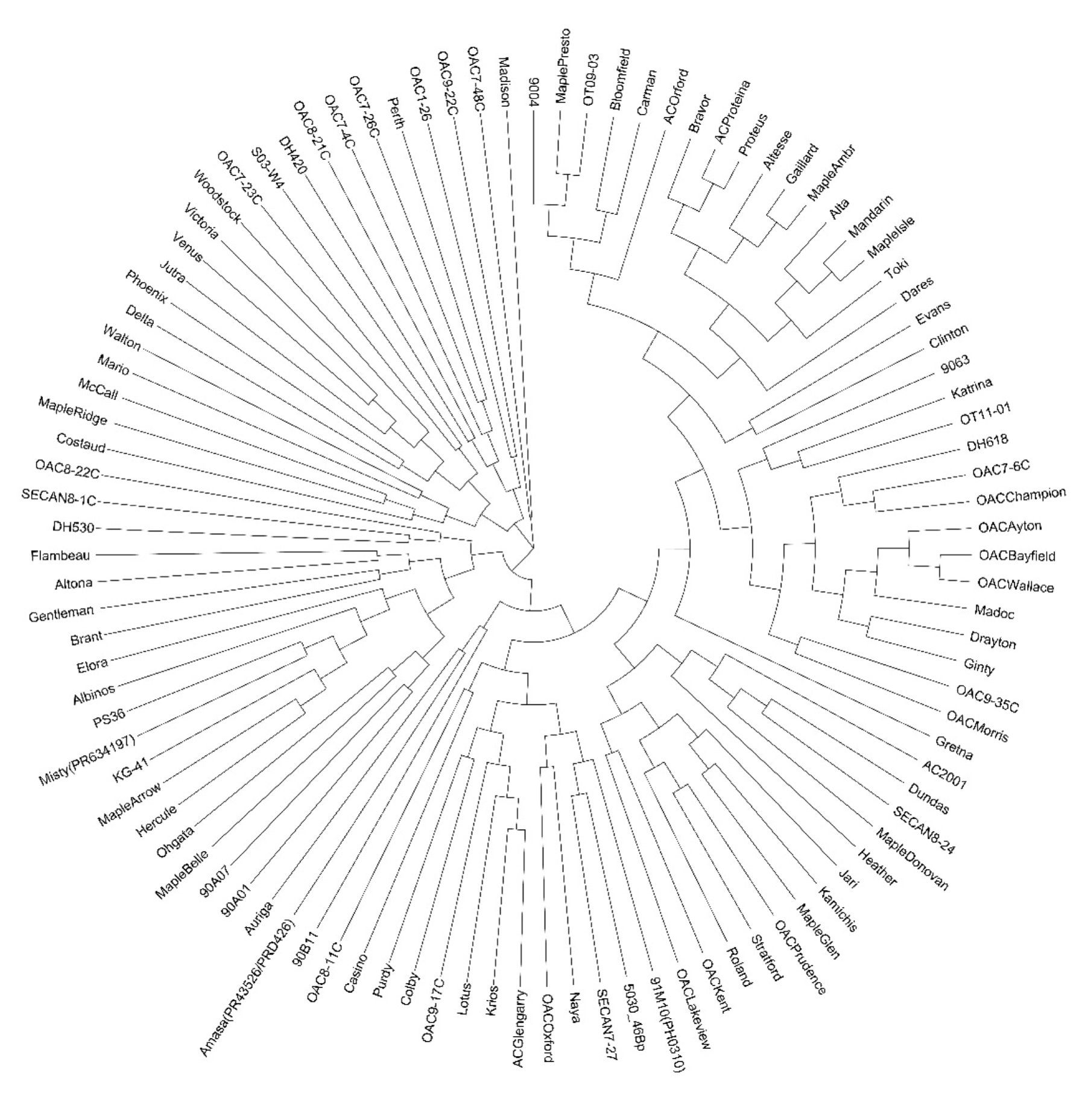

Supplement: Supplementary file 1 [file plants-12-01004-s001.zip › Supplementary Figure S1.tiff]

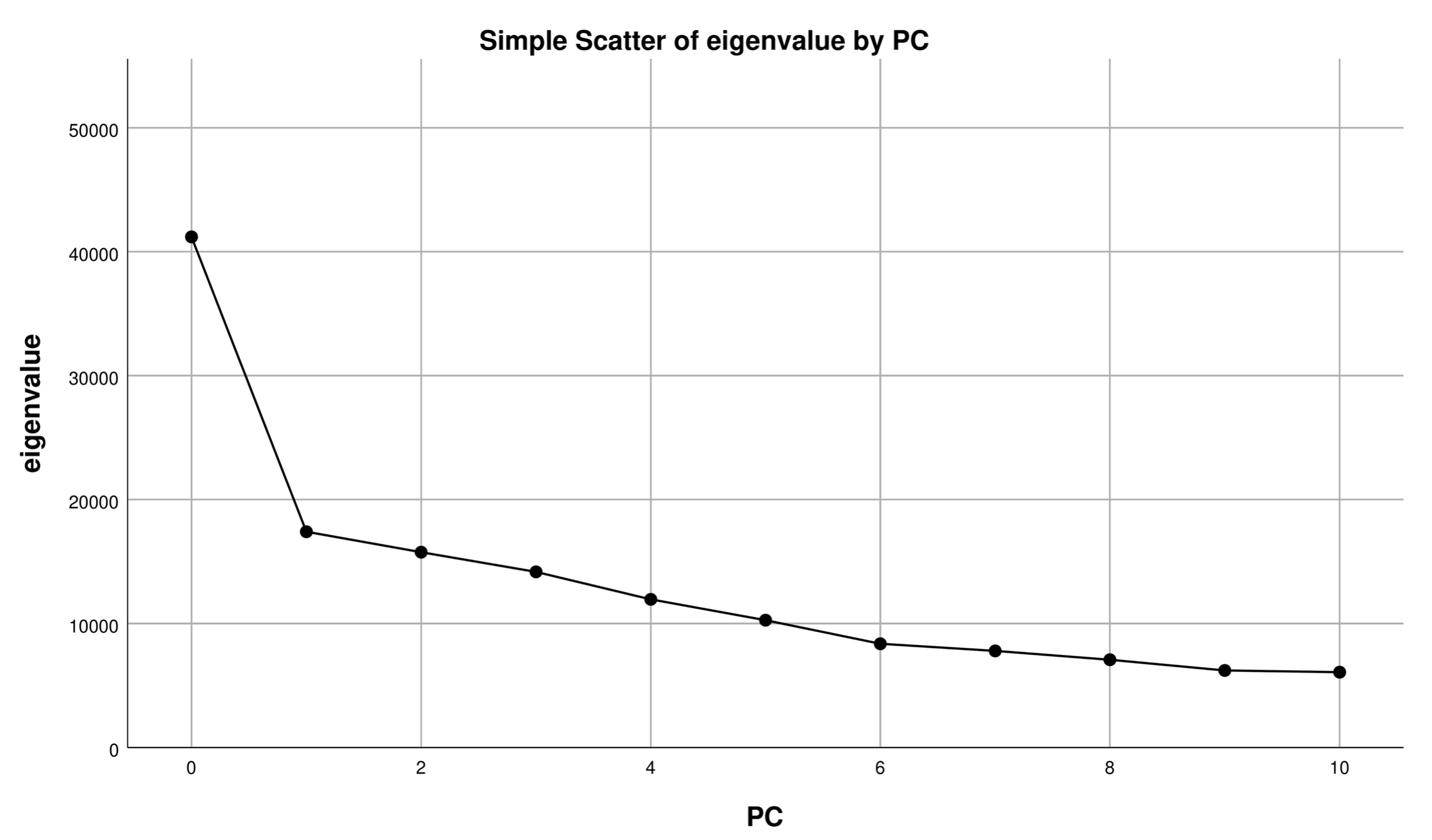

Supplement: Supplementary file 1 [file plants-12-01004-s001.zip › Supplementary Figure S2.tiff]

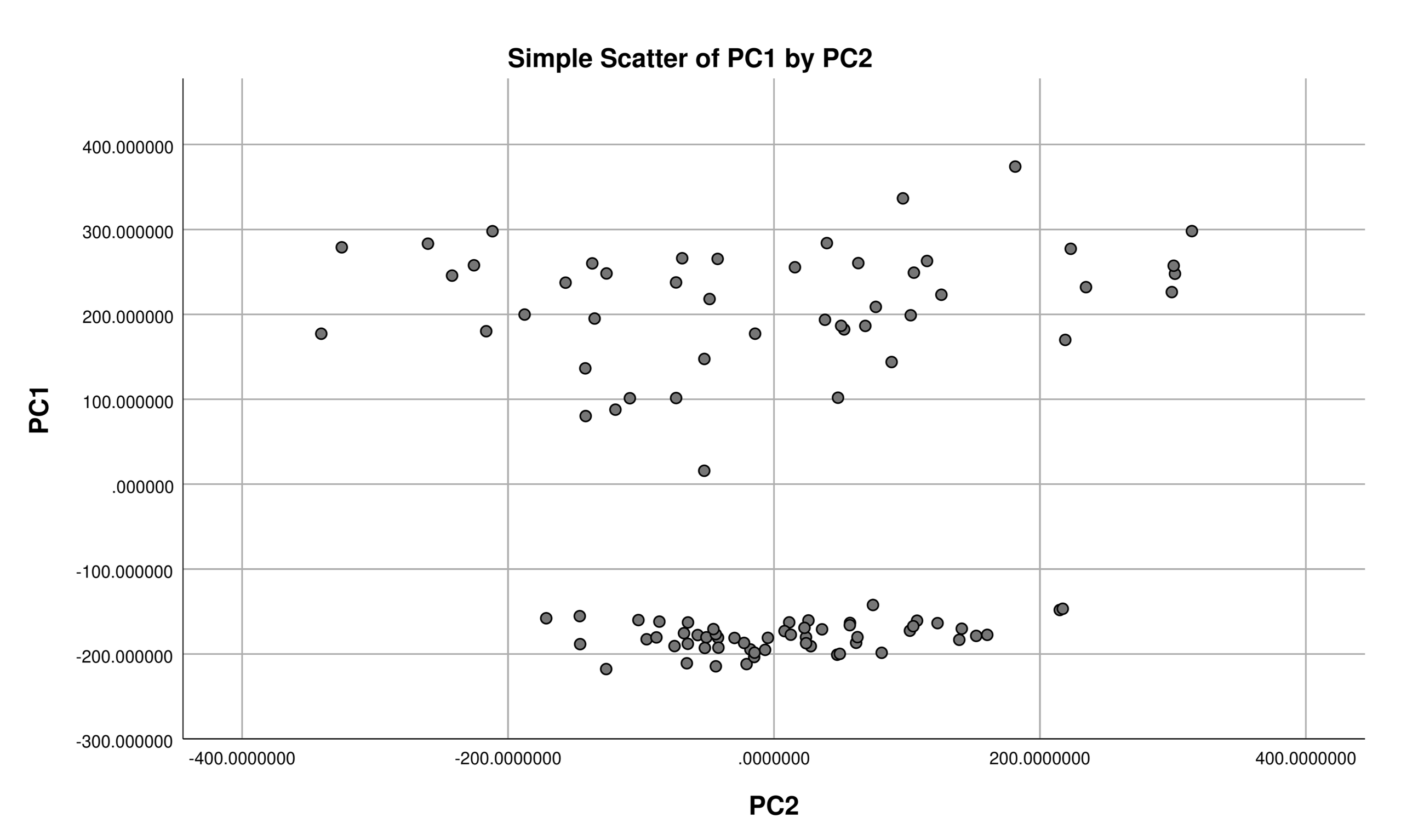

Supplement: Supplementary file 1 [file plants-12-01004-s001.zip › Supplementary Figure S3.tiff]

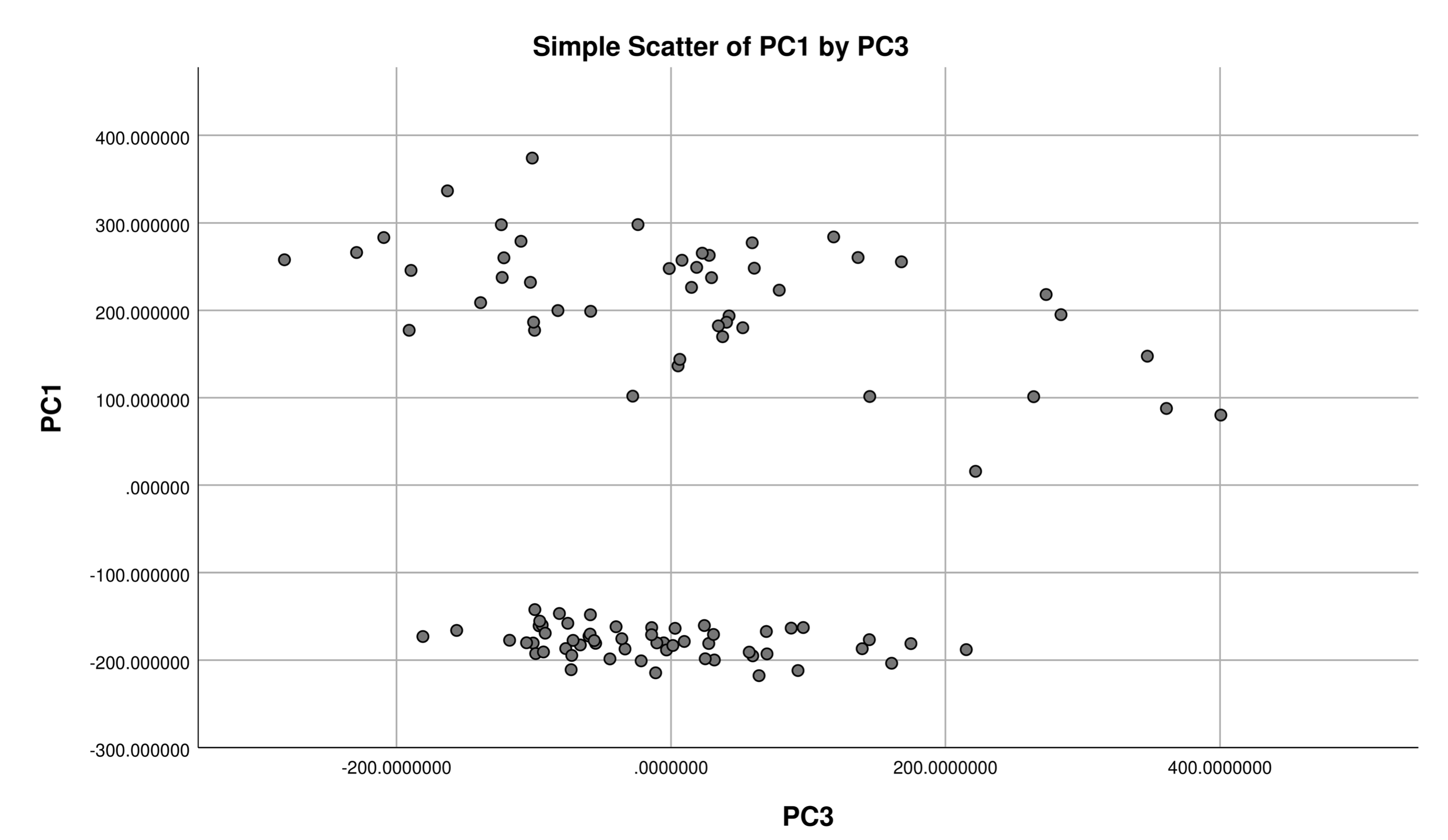

Supplement: Supplementary file 1 [file plants-12-01004-s001.zip › Supplementary Figure S4.tiff]

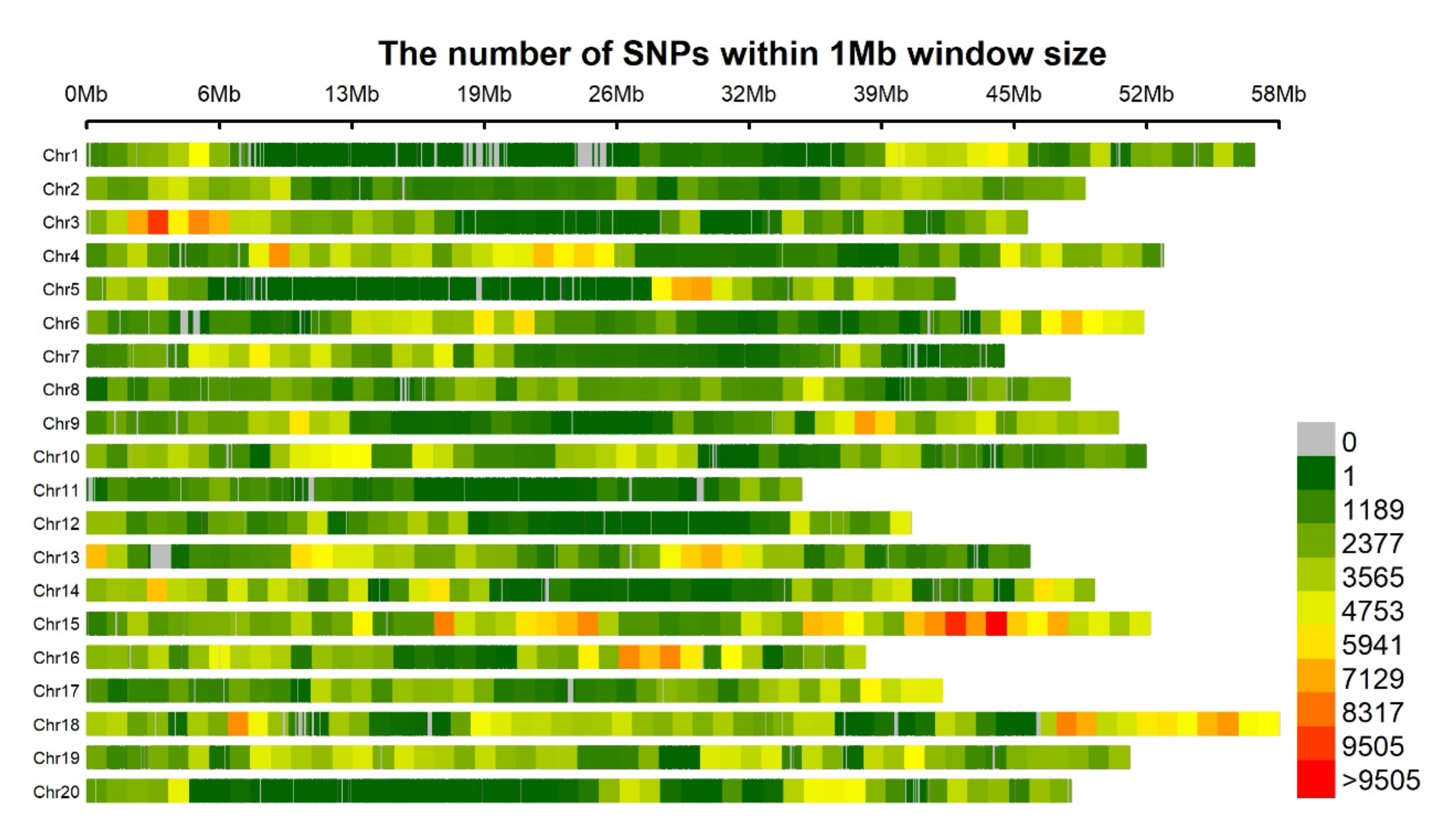

Supplement: Supplementary file 1 [file plants-12-01004-s001.zip › Supplementary Figure S5.tiff]
